# Factors Influencing the Statistical Power of Complex Data Analysis Protocols for Molecular Signature Development from Microarray Data

**DOI:** 10.1371/journal.pone.0004922

**Published:** 2009-03-17

**Authors:** Constantin F. Aliferis, Alexander Statnikov, Ioannis Tsamardinos, Jonathan S. Schildcrout, Bryan E. Shepherd, Frank E. Harrell

**Affiliations:** 1 Center of Health Informatics and Bioinformatics, New York University, New York, New York, United States of America; 2 Department of Biomedical Informatics, Vanderbilt University, Nashville, Tennessee, United States of America; 3 Department of Biostatistics, Vanderbilt University, Nashville, Tennessee, United States of America; 4 Department of Computer Science, University of Crete, Iraklio, Greece; University of the Western Cape, South Africa

## Abstract

**Background:**

Critical to the development of molecular signatures from microarray and other high-throughput data is testing the statistical significance of the produced signature in order to ensure its statistical reproducibility. While current best practices emphasize sufficiently powered univariate tests of differential expression, little is known about the factors that affect the statistical power of complex multivariate analysis protocols for high-dimensional molecular signature development.

**Methodology/Principal Findings:**

We show that choices of specific components of the analysis (i.e., error metric, classifier, error estimator and event balancing) have large and compounding effects on statistical power. The effects are demonstrated empirically by an analysis of 7 of the largest microarray cancer outcome prediction datasets and supplementary simulations, and by contrasting them to prior analyses of the same data.

**Conclusions/Significance:**

The findings of the present study have two important practical implications: First, high-throughput studies by avoiding under-powered data analysis protocols, can achieve substantial economies in sample required to demonstrate statistical significance of predictive signal. Factors that affect power are identified and studied. Much less sample than previously thought may be sufficient for exploratory studies as long as these factors are taken into consideration when designing and executing the analysis. Second, previous highly-cited claims that microarray assays may not be able to predict disease outcomes better than chance are shown by our experiments to be due to under-powered data analysis combined with inappropriate statistical tests.

## Introduction

Microarrays and other high-throughput assaying technologies have generated immense opportunities for discovery spanning the spectrum from basic research to clinical studies [1]–[3]. As the field moves from simpler analyses (e.g., differential expression of single genes and clustering) to more complex analyses such as developing multivariate molecular signatures in supervised fashion, the interpretation of microarray data involves multifaceted analysis protocols with many sophisticated and interacting analytic steps [4]. Developing molecular signatures in particular, is playing an increasingly important role in a variety of research design objectives both in basic and translational studies. Such objectives include, for example, detecting complex and coordinated patterns of transcriptional response to chemotherapeutic agents on cell lines and predicting subsequent patient treatment response on the basis of this information [5], discovery of new drug targets [6], discovery of biomarkers [7], subtyping diseases [8] and personalizing treatments [9].

The reproducibility of gene expression microarrays across laboratories for individual gene expression measurements and the ability to differentiate between disease subtypes are well established in recent studies [2], [10], [11]. Essential to developing molecular signatures is not only assay reproducibility however, but also *statistical* reproducibility. The latter can be directly assessed by tests of statistical significance of the produced signatures. These tests are usually permutation based and were introduced in bioinformatics by [12], [13] based on foundational works of [14], [15].

Although substantial efforts have been invested in studying the statistical power of differential gene expression [16], [17], much less is known currently about the power of testing molecular signatures for statistically significant (hence reproducible, “real”) signal. The present work shows that four specific components of data analysis (error metric, error estimator, classifier, and event balancing) have significant and compounding effects on probability (i.e., statistical power) to detect true signal in molecular signatures. These findings can help researchers design data analysis protocols that require fewer samples; they also shed light on the appropriateness of microarrays as an assay platform for outcome prediction. The present report uses theoretical analysis, simulation experiments, and empirical analysis of 7 human gene expression datasets [8], [9], [18]–[22]. The datasets were chosen so that the comparison to a previously published highly-cited protocol [23] constitutes a case study that demonstrates the practical benefits of improved statistical power on the resource efficiency and validity of analysis.

## Results

We start with a theoretical analysis that shows how the choice of four specific components of data analysis protocols for molecular signature development and their statistical testing affects the statistical power to detect predictive signal. We then present a simulation study that demonstrates that depending on choice of the above components even strong signals can fail to be detected with routine sample sizes and that the effects of each component on statistical power are large and compounded. We subsequently test the insights and hypotheses generated by the theoretical analysis and simulation studies with real gene expression data. Specifically, we analyze 7 datasets [8], [9], [18]–[22]. These datasets are important for two reasons: first, they have been used to derive both clinically relevant signatures and to investigate underlying biological processes [8], [9], [18], [20]–[22], [24]. Second, a highly-cited prior analysis of the same datasets [23] reached the conclusion that statistically significant signal cannot be detected in 5 out of 7 datasets and thus either microarrays are incapable of predicting clinical outcomes (and by extension, giving insight into the biology of disease progression) or that studies with a few hundred samples are insufficient and only sample sizes in the order of thousands will lead to statistically reproducible findings [25], [26]. Our investigation into the factors that affect power allows us to test the hypothesis that these prior results were due in large part to an underpowered analysis protocol, showing thus the great importance of careful planning of data analytics with an eye toward sufficient power.

### Theoretical analysis

#### Effects of error metric on power

Fundamental to the assessment of predictive signal of molecular signatures is the choice of error metric that is used to quantify predictivity. An unfortunate frequent practice in the field of bioinformatics to date is to use as classification performance metric the proportion of misclassifications. Discontinuous error metrics such as proportion of misclassifications, sensitivity, and specificity are “improper scoring rules” however, since they impose arbitrary thresholds on predictor models and do not capture the uncertainty in the predictions [27]. The proportion of misclassifications moreover, is known to yield estimators with low power to detect signals in data when compared to other metrics such as area under the receiver operating characteristic (ROC) curve (AUC) [27]. The ROC curve is the plot of sensitivity versus 1-specificity for a range of continuous or discrete classification threshold values. AUC is equivalent to a rank correlation between predicted outcome probability and the observed outcome, requiring no categorization. AUC ranges from 0 to 1, with an AUC equal to 0 indicating the worst possible classifier, 0.5 representing a random (i.e., uninformative) classifier, and 1 representing perfect classification. Testing whether predictions are unrelated to true outcomes using AUC is equivalent to the Wilcoxon test, while testing for proportion of misclassifications is equivalent to using the Mood median test which has been shown to have poor efficiency compared to the Wilcoxon test [28]. A broader, non-parametric justification why AUC is more discriminative than proportion of misclassifications is provided by [29]. Supporting Information File S1 provides an example where two signatures have the same proportions of misclassifications but different predictivity which is captured by the AUC metric. Although counter-examples do exist, they are relatively rarer [29]. Hence the AUC is more powerful than proportion of misclassifications.

#### Effects of classifier on power

Statistical power is increased whenever the tested effect size (predictivity in our context) is larger and the variance is smaller (assuming fixed sample size for simplicity). Hence using a classifier that produces the most predictive signature (everything else being equal) directly translates to improved statistical power for detecting predictive signal. Statistical machine learning theory proves that different classifiers have different inductive biases (i.e., preferences for classes of models), and that a classifier family has to be matched to the characteristics of the domain in order to achieve optimal predictivity (and correspondingly optimal power to detect signal) [30]. Indeed, recent empirical studies with gene expression data have shown that specific classifiers, such as Support Vector Machines (SVMs) produce models with stronger predictive ability (signal) and higher robustness across many high-throughput datasets compared to several widely-used alternatives [31]–[33]. Other authors also corroborate the need to choose classifiers carefully by recommending against some complex classifiers in order to avoid overfitting [4]. The above results have been neglected by some authors [23] who claim that “*in principle, there is no biological or mathematical reason why one particular classification method should be better than others*” and do not optimize the choice of classifier for the data at hand when conducting statistical testing of microarray gene expression signatures. We will show that this adversely affects the power of their analyses.

#### Effects of error estimator on power

Procedures that estimate the generalization error of a signature are called “error estimators”. A commonly used estimator is the *holdout estimator*. The holdout estimator is based on splitting the data in two random non-overlapping parts, deriving a signature from the first one and assessing its error in the second one. The holdout estimator is asymptotically unbiased, that is, with infinite test sample it produces an estimate that is the true error in the population. In small samples holdout estimates often deviate from the large-sample value. This variability is reduced as sample size grows [34].

From standard power-size analysis considerations it follows that the lowest-variance unbiased estimator has highest power [35]. Unfortunately, the holdout estimator has larger variance compared to several other unbiased estimators used in molecular signature studies [34], and this naturally leads to reduced statistical power. We elaborate on the reasons for this behavior by comparing the holdout to the *repeated 10-fold cross-validation estimator* [36]. The latter estimator is a variant of the well-known 10-fold cross-validation estimator which is calculated by balanced splitting of the data into 10 non-overlapping sample sets used for testing (while each complementing set is used for training) and averaging the test errors. The repeated 10-fold cross-validation estimator is obtained by running regular 10-fold cross-validation for 100 (or other sufficient number of) times with different splits of data into training and testing sets each time and by reporting the average estimate over all runs.

To see why holdout has higher variance than repeated 10-fold cross-validation, consider that there are several major sources of variance of estimators in practical use. These are: *sampling variance*, *split variance*, *testing set size*, and *internal variance*. Sampling variance refers to the uncertainty associated with drawing a random sample of fixed finite size from a population. Split variance refers to uncertainty associated with drawing a random split of training and testing sets from all possible splits of a given sample with fixed training-testing ratio. Testing set size variance refers to variability in error estimates due to finite testing dataset size. Finally, internal variance refers to uncertainty associated with classifier instability (i.e., different training datasets lead to different signatures) and increases as training set size decreases [30], [36]. The repeated 10-fold cross-validation essentially eliminates split variance by using many splits and averaging over them, and furthermore it reduces internal variance by using more sample for training than holdout (under typical training-testing split ratios). Both estimators have the same sampling variance. Finally, while testing set size variance is larger in the repeated 10-fold estimator than the holdout, the combination of higher split and internal variance makes overall the holdout to have higher variance and to be less powerful than repeated 10-fold cross-validation in many practical situations. Unfortunately this is often neglected in practical analysis and therefore using the holdout estimator leads to reduced ability to establish statistical significance of signatures.

#### Effects of event balancing on power

When in the context of error estimation one enforces that both the training and testing sets have the same proportion of events and non-events as the original full data, we will call such error estimation “event balanced”. An important and subtle shortcoming of some data analysis protocols is to not balance the training and testing data, seriously affecting variance, statistical power (and potentially biasing error estimates). For example, in [23] the models were trained on samples with 50% event rates. They were then tested on samples the event prevalence of which was far below 50% thus yielding estimates that were less efficient than the standard holdout estimator in which the data are split at random. The result of this is evident in Figure 2 from [23] in which as the sampling moves to larger training sets, this forces the testing sets in addition to being smaller, to implicitly have a very low event rate and thus large variance of error estimates. Notice that most classifiers, including the one used by [23], are designed to work under the assumption that the training and testing sets are identically distributed [30]. It is thus unrealistic to expect in general that a classifier that is trained using data from a distribution where events and non-events are equally likely will perform well, without adjustments [37], in a different distribution where this ratio is heavily distorted. This is especially so when using an error metric that is sensitive to event priors such as proportion of misclassifications. Supporting Information File S2 shows via an example that this shift in distributions can affect the performance of even an optimal classifier, i.e., one that has learned perfectly the distribution of the training data, to the point of appearing to be no better than flipping a coin.

### Simulation experiments

A primary purpose of the simulation experiments is to demonstrate and study the relative importance of the above factors that are hypothesized on the previous theoretical grounds to influence power of complex data analysis of high-throughput data. The simulation uses an idealized analysis in which the data-generating process is known and the true moderate-strength signal is present even in small samples. Such analysis is typical in literature discussing statistical issues surrounding microarray data because knowing the generative model allows a precise characterization of the strengths and limitations of data analysis techniques [38]. Details of the simulation are provided in the Supporting Information File S3. A second goal is to examine the statistical power of a previously published data analysis protocol [23] (“Protocol I”, that employs non-balanced holdout estimator with proportion of correct classifications and a nearest-centroid classifier), specifically when varying these four factors. Finally, a third goal is to test the relative power of the theoretically expected more powerful protocol (“Protocol II”, that employs balanced repeated 10-fold cross-validation estimator with AUC as the error metric and SVMs as classifier). The best protocol in the simulations will then be validated in the next sub-section with real data.

**Figure 1:**
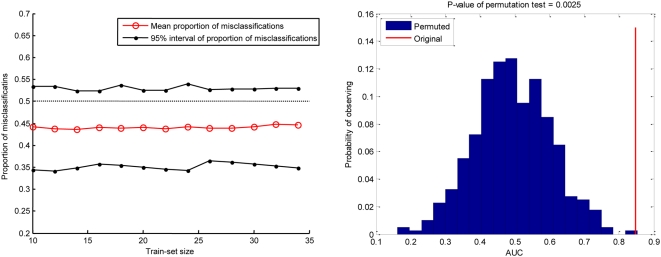
Comparison of Protocols I and II in simulated data. *Left:* Example where the Protocol I [23] applied to simulated data with true moderate-strength signal fails to detect statistical significance at all training set sizes. *Right*: a more powerful protocol (Protocol II, based on event balanced repeated 10-fold cross-validation with SVM classifiers and AUC metric) detects statistically significant predictive signal according to an outcome-value permutation test. Specifically, the p-value of the null hypothesis of no signal is 0.0025. The blue bars depict the distribution of repeated 10-fold cross-validation AUC estimates over 400 random datasets produced via outcome value permutation. The red line depicts the value of repeated 10-fold cross-validation AUC on the original data (i.e., without perturbing the outcome values).

The left part of Figure 1 demonstrates the inability of Protocol I [23] to detect signal which is detectable by Protocol II. The right part of this figure shows results of application of Protocol II and assessment of its statistical significance by permutation testing (details about statistical significance testing are provided in the Materials and Methods section). Overall Protocol I has remarkably small power ranging from less than 0.002 to 0.3 (depending on the criterion used for rejecting the null hypothesis, please see Supporting Information File S3). In contrast, Protocol II has power 0.93. By replacing proportion of misclassifications with AUC in Protocol I, its power increases to 0.6, and by additionally adding the use of SVMs, it further increases to 0.75. Conversely, if we start with Protocol II and replace AUC with proportion of misclassifications and SVMs with the classifier from [23], these changes reduce the power from 0.93 to 0.46. These empirical power estimates do not provide the exact power in real datasets since the true nature of the corresponding distributions is not known and varies among datasets. However the simulation strengthens our hypothesis that the choice of error metric, classifier, event balancing and error estimator have large impact on study results and sheds light on the limitations of the analyses described in prior work [23]. In the next sub-section we test the Protocol II in real data (where Protocol I was previously independently applied).

### Analysis of real gene expression data

**Figure 2:**
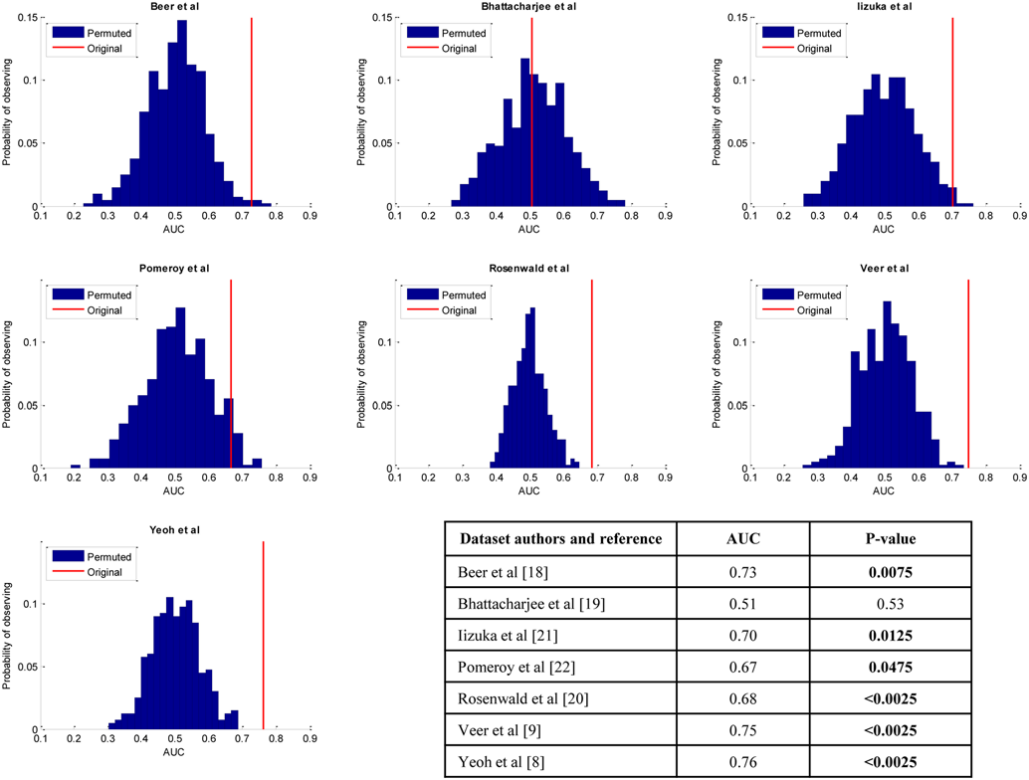
Application of Protocol II to human microarray data. Each histogram is the distribution of the repeated 10-fold cross-validation AUC estimates for each dataset under the null hypothesis “there is no signal present in the data” (as computed by 400 random outcome value permutations). The red line in each graph is the observed value of AUC estimated by the repeated 10-fold cross-validation on the original data. AUC and p-values are shown for each dataset in the embedded table. Bold p-values indicate that the null hypothesis is rejected at the 0.05 level in these datasets.

Figure 2 reports the AUC estimates produced with Protocol II for each one of the 7 real datasets along with p-values for testing the null hypothesis that the produced signatures are uninformative (i.e., with no signal). As can be seen in Figure 2, statistically significant signal (at the 0.05 level) can be detected in 6 out of 7 datasets compared to 2 out of 7 in the prior study that had used the less powerful Protocol I [23]. The p-values are calculated by a standard label-permutation procedure (see Materials and Methods section). The histograms in Figure 2 depict with blue the distribution of the repeated 10-fold cross-validation AUC estimates from Protocol II for datasets produced under the null hypothesis of “no predictive signal” and with red the repeated 10-fold cross-validation AUC estimates from Protocol II for the original datasets.

The above repeated 10-fold cross-validation AUC estimates in the datasets that had statistically significant signal ranged from 0.67 to 0.76, indicating that even signal with weak strength can be shown in real data to be statistically significant with moderate sample sizes. The U-statistic based confidence intervals for repeated 10-fold cross-validation AUC estimates are provided in the Supporting Information File S4 and they are consistent with the above conclusions. Notice also that under the null hypothesis of no predictive signal the distribution of the repeated 10-fold cross-validation AUC estimates is centered at 0.5, which corroborates the theoretical expectation that the error estimates are unbiased and that Protocol II does not overfit (more details in the Supporting Information File S5).

#### A note on the choice of null hypothesis for statistical significance testing

The combined simulated and real data results show very significant differences in the ability of Protocol I and II to detect real signal. This prompted us to investigate further the underlying differences between the two protocols. An unanticipated finding was that a major discrepancy between the two protocols exists in the precise null hypothesis tested: Ideally, one wishes to test the broad null hypothesis “there is no signal in the data”. Rejecting this hypothesis entails that the observed signal in the sample will generalize in the population where the sample is drawn from. There exist several reasons why an observed signal may not be present in the population. First, the available sample may be non-representative of the population. Another reason is that a splitting procedure of the sample into training and testing parts may yield non-representative training or testing datasets (we will refer to this as “bad” split of the data). The previously published procedure for statistical significance testing of the signatures of Protocol I (see Materials and Methods section) eventually tests for only one source of non-reproducible signal: “bad” split of the sample data (and also conducts a sensitivity analysis on the training sample size). If this procedure instead was using sampling with replacement, it would amount to a simple Bootstrap estimator and thus would test for a non-representative sample as well. However because the Bootstrap introduces a bias in the error estimates that is difficult to correct, the above procedure samples without replacement and tests only for a restricted null hypothesis (i.e., “bad” data split). In contrast, the statistical significance procedure utilized by Protocol II (see Materials and Methods section) uses a repeated split cross-validation estimator effectively eliminating uncertainty introduced by non-representative splits. In addition by permuting labels, Protocol II effectively samples from a population where the gene expression patterns as well as the event rate are fixed, and there is no relationship between gene expression patterns and outcome (hence it is equivalent to the null hypothesis of no signal in the population). Under this label permuting any apparent relationship between gene expression patterns and outcomes is due to sampling variation. Thus Protocol II tests for a much more informative null hypothesis than the statistical test in Protocol I. Notice that the four factors affecting power we identified earlier affect both null hypotheses and have noteworthy effects on both protocols as shown in the simulation studies. The null hypothesis tested by the test of significance of Protocol I is too limited and redundant (i.e., as long as a repeated split cross-validation estimator is used) and should not be pursued in practice. However because of the broad implications previously drawn by applying Protocol I, it was necessary to test it in the present study in order to precisely identify the reasons why this protocol failed to establish signal in real microarray datasets.

## Discussion

The present work shows that several important components of data analysis for molecular signature creation have significant and compounding effects on probability to detect true signal (i.e., statistical power). Four factors (choice of error metric, classifier, error estimator, and event balancing) were investigated by theoretical assessment, simulation study, and application to 7 human microarray datasets.

Our findings indicate that the choices made in the data analysis protocol corresponding to the four factors studied can improve power and by extension research efficiency. Increasing study sample size (as for example proposed by [26]) increases statistical power, but also dramatically increases study costs and delays study completion. In contrast, application of efficient statistical protocols has the potential to significantly improve the chances of detecting real signal with modest sample sizes. Conversely, even very large samples can be “wasted” when analyzed with under-powered (i.e., inefficient) data analysis procedures.

Our data also shows clearly that the highly-cited study [23] that concluded that “*Five of the seven largest published studies addressing cancer prognosis did not classify patients better than chance*” reached these conclusions because of two main reasons: first, the specific null hypothesis tested was inappropriate and second, because several underpowered analysis components were employed. These two reasons were inextricably intertwined in the data analysis protocol employed. The ensuing controversy in the field of disease outcome prediction using microarrays seems thus to be an artifact of data analysis and not an intrinsic limitation of this assaying technology. The present findings therefore have direct positive implications for the feasibility of related research in new drug development, personalizing treatments and adapting clinical trials to patient genomic characteristics. Inappropriate data analysis methodology can create a climate of distrust about the underlying assay technology and findings that may lead to wasteful development processes. For example in our assessment, using a series of datasets for validation [39]–[42] likely wastes time and money with no substantial benefit. Validation using a single independent dataset from the same population of patients as used for construction of the signature is sufficient if the protocols used are unbiased and appropriately powered.

We note that it is possible that gene selection and better optimization/choice of classifiers could achieve predictivity and power improvements over the protocol used in the present paper [33]. For example, gene selection and error estimation using the more sophisticated but computationally more demanding nested cross-validation designs [43] was not pursued in order to keep the computational requirements of running extensive permutation tests under control.

We finally observe that the factors studied have been the subject of substantial prior research in biostatistics and bioinformatics. However their relationship to statistical power for molecular signature testing has not been systematically investigated previously. For example, recent work has proposed a much-needed and comprehensive set of guidelines for the analysis and reporting of microarray and other “omics” data [4]. However the choice of classifier is not addressed as of crucial importance, the choice of error metric and estimator is not linked to statistical power, and event balancing as a source of bias and low power is not addressed. These omissions demonstrate the subtle effects of these factors on statistical power and that these effects have gone largely unnoticed in the field so far.

In conclusion, factors that affect the statistical power of complex analysis protocols for molecular signature development from high-throughput data constitute an important area for study. The present paper showed that choices of error metric, classifier, error estimator and event balancing have large and compounding effects on statistical power. They can further be combined with inappropriate null hypotheses to yield ineffective analysis protocols. An experimental comparison of data analysis protocols reveals that previous highly-cited claims that microarray assays may not be able to predict clinical outcomes better than chance are byproducts of data analysis limitations. Research designs of high-throughput studies will benefit by using the most powerful data analysis protocols available combined with appropriate statistical tests and doing so leads to substantial economies of required sample. New data analysis protocols should be tested for statistical efficiency before deploying for building molecular signatures. We recommend testing against existing protocols (such as one presented in this paper) in simulated or real data with known predictive signal using datasets in which the experimenter varies sample sizes [44], [45].

## Materials and Methods

### Microarray Datasets

The characteristics of the human datasets analyzed [8], [9], [18]–[22] are summarized in Table 1.

**Table 1:** Characteristics of gene expression microarray datasets analyzed in this study.

Dataset authors and reference	Sample size and number of events	Number of variables (genes)	Predicted event (outcome)
Beer et al [18]	**86** (24 events)	**7129**	Lung adenocarcinoma survival
Bhattacharjee et al [19]	**62** (31 events)	**12600**	Lung adenocarcinoma 4-year survival
Iizuka et al [21]	**60** (20 events)	**7070**	Hepatocellular carcinoma 1-year recurrence-free survival
Pomeroy et al [22]	**60** (21 events)	**7129**	Medulloblastoma survival
Rosenwald et al [20]	**240** (138 events)	**7399**	Non-Hodgkin lymphoma survival
Veer et al [9]	**97** (46 events)	**24188**	Breast cancer 5-year metastasis-free survival
Yeoh et al [8]	**233** (32 events)	**12240**	Acute lymphocytic leukemia relapse-free survival

### Error / prediction performance metric

The area under the receiver operating characteristic (ROC) curve (AUC) is calculated by the formula provided in [46]. Proportion of misclassifications is calculated as the ratio: number of wrong classification divided by total number of classifications.

### Classifiers & gene selection

Protocol I [23] involves selection of 50 genes with the highest correlation in training data with outcome variable according to Pearson's correlation coefficient. Then molecular signatures are developed based on these genes using a nearest-centroid prediction method [47].

Protocol II uses the LibSVM implementation of Support Vector Machines (SVMs) to build molecular signatures with a fixed misclassification penalty parameter *C* = 100, and a linear kernel [48]. Gene selection is not employed to avoid increased computational costs. We note that SVMs have built-in regularization however, which means that the learning algorithm penalizes large weights of predictors thus favoring simpler models by implicitly selecting genes, without using explicit gene selection procedures [49], [50].

### Statistical analysis

Statistical significance of the molecular signatures in Protocol I replicates the procedure of [23]. Namely, 500 training datasets of size *n* are obtained by sampling without replacement the original dataset (of size *N*) such that each training set has *n*/2 subjects with each outcome. For each training set, the testing set is defined as its complement (of size *N-n*). The molecular signatures are then fitted on the training sets and their classification performance is assessed on the corresponding testing sets. The above procedure is repeated for different training set sizes ranging from 10 to a maximum value which was chosen so that the testing set has at least one subject representing each outcome. Given a distribution of classification performances for each training set size, the corresponding 95% intervals are constructed. The original dataset is considered to contain predictive signal if the upper 95% interval limit is less than 0.5 proportion of misclassifications. Notice that the published description of this method [23] does not explicitly state whether the above condition for significance should hold in at least one training set size *n*, or all possible training set sizes, or the majority of them. Thus we examine all three possibilities in the present work.

For Protocol II, we use outcome value-permutation to test in each dataset the null hypothesis of no predictive signal [12], [13]. This is also known as a randomization test or a Monte-Carlo permutation test. We construct the distribution corresponding to the null hypothesis by randomly permuting the values of the outcome variable (400 times) and then using SVMs (as described above) to compute the signature and repeated 10-fold cross-validation estimate of AUC for each permuted dataset. The repeated 10-fold cross-validation estimate from the original data is then compared to this distribution, and p-values correspond to the proportion of permuted estimators (under the null hypothesis) that are more extreme than the repeated 10-fold cross-validation estimate from the original (non-outcome value permuted) data.

## Supporting Information

File S1 Comparison of proportion of misclassifications with area under ROC curve (AUC)
(0.06 MB DOC)

File S2 Demonstration of pitfalls of non-balanced data
(0.10 MB DOC)

File S3 Details of simulation experiments
(0.08 MB DOC)

File S4 Confidence intervals for repeated 10-fold cross-validation AUC estimates
(0.08 MB DOC)

File S5 Demonstration that Protocol II is not biased
(0.09 MB DOC)
