# Supplementary material for: Factors Influencing the Statistical Power of Complex Data Analysis Protocols for Molecular Signature Development from Microarray Data
Source: PLoS One. 2009 Mar 17;4(3):e4922. doi: 10.1371/journal.pone.0004922 (PMC2654113; doi:10.1371/journal.pone.0004922)
Supplement: File S1 — Comparison of proportion of misclassifications with area under ROC curve (AUC) (0.06 MB DOC) [file pone.0004922.s001.doc]

***Supporting Information File S1:***

**Comparison of proportion of misclassifications with area under ROC curve (AUC)**

The proportion of misclassifications is an arbitrary error metric with low statistical power and thus with weak ability, compared to other metrics, to detect signal in the data as described in the main text. Figure S1 below demonstrates an example where two classifiers (i.e., molecular signatures) have the same proportion of misclassifications but different AUCs (thus, the AUC is more discriminative than the proportion of misclassifications). Although counter-examples do exist, they are relatively rarer [1].

**Figure S1**: The area under the ROC curve (AUC) is more discriminatory than proportion of misclassifications. In this example, classifiers A and B have equal proportions of misclassifications (0.6) but different AUCs (0.8 and 0.4). Also note that classifier C has proportion of misclassifications consistent with being uninformative, but it is highly informative.

**References**

1. Ling CX, Huang J, Zhang H (2003) AUC: a statistically consistent and more discriminating measure than accuracy. Proceedings of the Eighteenth International Joint Conference of Artificial Intelligence (IJCAI)
