# Supplementary material for: Factors Influencing the Statistical Power of Complex Data Analysis Protocols for Molecular Signature Development from Microarray Data
Source: PLoS One. 2009 Mar 17;4(3):e4922. doi: 10.1371/journal.pone.0004922 (PMC2654113; doi:10.1371/journal.pone.0004922)
Supplement: File S2 — Demonstration of pitfalls of non-balanced data (0.10 MB DOC) [file pone.0004922.s002.doc]

***Supporting Information File S2:***

**Demonstration of pitfalls of non-balanced data**

Balancing the training data without balancing the testing data of the holdout estimator or adjusting the classifier [1] yields a biased error estimator and an under-powered test for statistical significance of the signal. Figure S2 provides a pictorial depiction of the splitting and why it affects classifiers adversely.

**Figure S2**. Left part: Consider the original ratio of events-to-non-events (denoted by gradated shading) which is 40%. Consider a training set of size 4 which will have 50% events according to the procedure of [2]. However, the corresponding testing set will have only 33.3% events (that is, it will be unbalanced). Such unbalancing corresponds to creating a different distribution and can affect adversely the holdout estimator’s variance. Right part: when training the same classifier in a different distribution than the one used to test it, even an optimal classifier may appear to have uninformative proportion of misclassifications. Furthermore, classifiers that are sensitive to the prior distribution of patterns and the class outcome values may be affected negatively.

**References**

1. Saerens M, Latinne P, Decaestecker C (2001) Adjusting the Outputs of a Classifier to New a Priori Probabilities: A Simple Procedure. Neural Computation 14: 21-41.

2. Michiels S, Koscielny S, Hill C (2005) Prediction of cancer outcome with microarrays: a multiple random validation strategy. Lancet 365: 488-492.
