# Supplementary material for: Factors Influencing the Statistical Power of Complex Data Analysis Protocols for Molecular Signature Development from Microarray Data
Source: PLoS One. 2009 Mar 17;4(3):e4922. doi: 10.1371/journal.pone.0004922 (PMC2654113; doi:10.1371/journal.pone.0004922)
Supplement: File S3 — Details of simulation experiments (0.08 MB DOC) [file pone.0004922.s003.doc]

***Supporting Information File S3:***

**Details of simulation experiments**

*Generation of simulated data*

Simulated data was generated as described below:

- **X** = matrix with gene values uniformly randomly distributed in the interval [0, 1] with *n* = 20,000 samples (rows) and *m* = 100 genes (columns).
- **E** = noise matrix initialized to uniformly randomly distributed values in the interval [0, 0.05] with *n* = 20,000 samples (rows) and *m* = 100 genes (columns).
- For *i* = 1,...*n* compute Y*_i_* = $\sum_{j=1}^{10} (\mathbf{X}_{i,j}+\mathbf{E}_{i,j})$, where $\mathbf{X}_{i,j}$ is the value of sample *i* of gene *j* and similarly for the noise matrix $\mathbf{E}_{i,j}$.
- Create the response variable R to be a thresholded function of Y by discretizing each value of the vector Y into binary so that 20% of the values are 1 and 80% are 0.
- Dataset for simulations = {R, **X**}.

*Classification error in simulated data as sample size increases*

As the sample size increases, the error is reduced for both the nearest-centroid classifier (as in Protocol I [1]) and the linear SVM classifier without gene selection (as in Protocol II). However the SVM classifier has a distinct power advantage over the nearest-centroid classifier. SVMs can detect the same predictive signal strength with less sample than the nearest centroid classifier.

Using the nearest centroid classifier:

| **Training set size** | **AUC metric** | **Proportion of misclassifications** |
| --- | --- | --- |
| 100 | 0.76 | 0.39 |
| 1,000 | 0.88 | 0.33 |
| 10,000 | 0.94 | 0.31 |

Using linear SVM classifier:

| **Training set size** | **AUC metric** | **Proportion of misclassifications** |
| --- | --- | --- |
| 100 | 0.81 | 0.19 |
| 1,000 | 0.98 | 0.07 |
| 10,000 | 0.99 | 0.03 |

*Empirical power over 500 random datasets of sample size 100*

In what follows we experiment with different combinations of error metric, classifier, error estimator, and event balancing in order to obtain estimates of the contribution of these factors on the ability to detect signal in the simulated data.

I. Using the classifier and gene selection method of [1]:

| **Method to detect signal** | | **Power (probability of detecting the (existing) signal)** | |
| --- | --- | --- | --- |
|  |  | Using AUC metric | Using proportion of misclassifications metric |
| **Method from** [1] | with detection rule “*signal for all sizes of the training set*” | <0.002 | <0.002 |
|  | with detection rule “*signal for at least one size of the training set*” | 0.60 | 0.30 |
|  | with detection rule “*signal for majority of sizes of the training set*” | 0.60 | 0.20 |
| **Repeated 10-fold cross-validation with permutation-based test to assess statistical significance** | | 0.78 | 0.46 |

Highlighted cells correspond to results obtained with Protocol I [1].

II. Using linear SVM classifier without gene selection:

| **Method to detect signal** | | **Power (probability of detecting the (existing) signal)** | |
| --- | --- | --- | --- |
|  |  | Using AUC metric | Using proportion of misclassifications metric |
| **Method from** [1] | with detection rule “*signal for all sizes of the training set*” | 0.002 | <0.002 |
|  | with detection rule “*signal for at least one size of the training set*” | 0.75 | 0.17 |
|  | with detection rule “*signal for majority of sizes of the training set*” | 0.62 | 0.01 |
| **Repeated 10-fold cross-validation with permutation-based test to assess statistical significance** | | 0.93 | 0.58 |

Highlighted cell corresponds to results obtained with Protocol II.

From the above it is clear that all of the studied factors (error metric, error estimator, event balancing, and choice of classifier) have large compounded effects on statistical power in accordance with theoretical expectations.

**References**

1. Michiels S, Koscielny S, Hill C (2005) Prediction of cancer outcome with microarrays: a multiple random validation strategy. Lancet 365: 488-492.
