# Supplementary material for: Factors Influencing the Statistical Power of Complex Data Analysis Protocols for Molecular Signature Development from Microarray Data
Source: PLoS One. 2009 Mar 17;4(3):e4922. doi: 10.1371/journal.pone.0004922 (PMC2654113; doi:10.1371/journal.pone.0004922)
Supplement: File S4 — Confidence intervals for repeated 10-fold cross-validation AUC estimates (0.08 MB DOC) [file pone.0004922.s004.doc]

***Supporting Information File S4:***

**Confidence intervals for repeated 10-fold cross-validation AUC estimates**

The 95% confidence intervals below were computed using a normal approximation based on U-statistic theory [1] and simulations involving the same number of events and non-events as in each dataset.

As it can be seen, the uninformative value of 0.5 AUC is not contained in the 95% confidence intervals for the true AUC (i.e., the predictive signal is statistically significantly different at the 0.05 level from no signal) in the same 6 out of 7 datasets as in the outcome value permutation analysis in the main text, further strengthening our belief in the statistical significance of these results.

| **Dataset authors and reference** | **AUC** | **95% confidence interval** |
| --- | --- | --- |
| Beer et al [2] | 0.73 | [0.61, 0.85] |
| Bhattacharjee et al [3] | 0.51 | [0.36, 0.65] |
| Iizuka et al [4] | 0.70 | [0.56, 0.84] |
| Pomeroy et al [5] | 0.67 | [0.52, 0.81] |
| Rosenwald et al [6] | 0.68 | [0.62, 0.75] |
| Veer et al [7] | 0.75 | [0.65, 0.85] |
| Yeoh et al [8] | 0.76 | [0.68, 0.85] |

**References**

1. Lee AJ (1990) U-statistics: theory and practice. New York: M. Dekker.

2. Beer DG, Kardia SL, Huang CC, Giordano TJ, Levin AM, et al. (2002) Gene-expression profiles predict survival of patients with lung adenocarcinoma. Nat Med 8: 816-824.

3. Bhattacharjee A, Richards WG, Staunton J, Li C, Monti S, et al. (2001) Classification of human lung carcinomas by mRNA expression profiling reveals distinct adenocarcinoma subclasses. Proc Natl Acad Sci U S A 98: 13790-13795.

4. Iizuka N, Oka M, Yamada-Okabe H, Nishida M, Maeda Y, et al. (2003) Oligonucleotide microarray for prediction of early intrahepatic recurrence of hepatocellular carcinoma after curative resection. Lancet 361: 923-929.

5. Pomeroy SL, Tamayo P, Gaasenbeek M, Sturla LM, Angelo M, et al. (2002) Prediction of central nervous system embryonal tumour outcome based on gene expression. Nature 415: 436-442.

6. Rosenwald A, Wright G, Chan WC, Connors JM, Campo E, et al. (2002) The use of molecular profiling to predict survival after chemotherapy for diffuse large-B-cell lymphoma. N Engl J Med 346: 1937-1947.

7. van't Veer LJ, Dai H, van de Vijver MJ, He YD, Hart AA, et al. (2002) Gene expression profiling predicts clinical outcome of breast cancer. Nature 415: 530-536.

8. Yeoh EJ, Ross ME, Shurtleff SA, Williams WK, Patel D, et al. (2002) Classification, subtype discovery, and prediction of outcome in pediatric acute lymphoblastic leukemia by gene expression profiling. Cancer Cell 1: 133-143.
