# Supplementary material for: Factors Influencing the Statistical Power of Complex Data Analysis Protocols for Molecular Signature Development from Microarray Data
Source: PLoS One. 2009 Mar 17;4(3):e4922. doi: 10.1371/journal.pone.0004922 (PMC2654113; doi:10.1371/journal.pone.0004922)
Supplement: File S5 — Demonstration that Protocol II is not biased (0.09 MB DOC) [file pone.0004922.s005.doc]

***Supporting Information File S5:***

**Demonstration that Protocol II is not biased**

One important component of a data analysis protocol is to test the error estimation for bias. We note that the repeated 10-fold cross-validation estimator just like every cross-validation estimator in practical use, is asymptotically unbiased under both the null (i.e., “no predictive signal is present”) and alternative hypotheses (i.e. “predictive signal is present”). Typically, the source of bias in protocols that use the repeated 10-fold cross-validation (or any other standard cross-validation) estimator is “contamination” of the model fitting in training datasets with information from testing datasets.

Under the assumption that contamination practices affect the null hypothesis at least as much or more than the alternative[[1]](#footnote-2), and since we cannot directly test for bias under the alternative hypothesis (because the true population error is not known), we test for bias under the null hypothesis (where the true population error *is* known). If therefore, a data analysis protocol using a cross-validation estimator is unbiased under the null hypothesis, this is strong evidence that it will also be unbiased under the alternative (because no contamination can be detected under the null hypothesis). An analogous argument applied to the context of study of how gene selection for the analysis of microarray data biases the holdout estimator is implicit in the simulations in [1].

Finally, establishing lack of bias using an outcome value permutation experiment establishes lack of bias in the sample size range that applies to our experiments, as opposed to asymptotic unbiasedness.

The procedure explained in [2,3] is based on obtaining multiple outcome value-permuted datasets, analyzing each dataset to build a classifier and estimate its generalization error, then testing this distribution against the theoretically expected AUC of 0.5 corresponding to the hypothesis of “no bias”. Here are results for Protocol II in 7 human gene expression datasets analyzed in this paper:

| **Dataset authors and reference** | **Mean AUC over 400 permutations of the outcome variable** | **95% confidence interval** |
| --- | --- | --- |
| Beer et al [4] | 0.50 | [0.31, 0.70] |
| Bhattacharjee et al [5] | 0.51 | [0.30, 0.70] |
| Iizuka et al [6] | 0.50 | [0.29 0.69] |
| Pomeroy et al [7] | 0.50 | [0.30, 0.70] |
| Rosenwald et al [8] | 0.50 | [0.41 0.60] |
| Veer et al [9] | 0.50 | [0.34, 0.66] |
| Yeoh et al [10] | 0.50 | [0.36, 0.65] |

As can be seen from the table, Protocol II provides unbiased point estimates averaged over all datasets. 95% confidence intervals of AUC contain the uninformative value of 0.5 AUC, indicating thus that we do not reject the hypothesis of “unbiased protocol”.

**References**

1. Simon R, Radmacher MD, Dobbin K, McShane LM (2003) Pitfalls in the use of DNA microarray data for diagnostic and prognostic classification. J Natl Cancer Inst 95: 14-18.

2. Aliferis CF, Statnikov A, Tsamardinos I (2006) Challenges in the analysis of mass-throughput data: a technical commentary from the statistical machine learning perspective. Cancer Informatics 2: 133-162.

3. Aliferis CF, Tsamardinos I, Massion PP, Statnikov A, Hardin D (2003) Why classification models using array gene expression data perform so well: a preliminary investigation of explanatory factors. Proceedings of the 2003 International Conference on Mathematics and Engineering Techniques in Medicine and Biological Sciences (METMBS)

4. Beer DG, Kardia SL, Huang CC, Giordano TJ, Levin AM, et al. (2002) Gene-expression profiles predict survival of patients with lung adenocarcinoma. Nat Med 8: 816-824.

5. Bhattacharjee A, Richards WG, Staunton J, Li C, Monti S, et al. (2001) Classification of human lung carcinomas by mRNA expression profiling reveals distinct adenocarcinoma subclasses. Proc Natl Acad Sci U S A 98: 13790-13795.

6. Iizuka N, Oka M, Yamada-Okabe H, Nishida M, Maeda Y, et al. (2003) Oligonucleotide microarray for prediction of early intrahepatic recurrence of hepatocellular carcinoma after curative resection. Lancet 361: 923-929.

7. Pomeroy SL, Tamayo P, Gaasenbeek M, Sturla LM, Angelo M, et al. (2002) Prediction of central nervous system embryonal tumour outcome based on gene expression. Nature 415: 436-442.

8. Rosenwald A, Wright G, Chan WC, Connors JM, Campo E, et al. (2002) The use of molecular profiling to predict survival after chemotherapy for diffuse large-B-cell lymphoma. N Engl J Med 346: 1937-1947.

9. van't Veer LJ, Dai H, van de Vijver MJ, He YD, Hart AA, et al. (2002) Gene expression profiling predicts clinical outcome of breast cancer. Nature 415: 530-536.

10. Yeoh EJ, Ross ME, Shurtleff SA, Williams WK, Patel D, et al. (2002) Classification, subtype discovery, and prediction of outcome in pediatric acute lymphoblastic leukemia by gene expression profiling. Cancer Cell 1: 133-143.

1. This is because, as the true signal grows, the margin for overfitting shrinks. Thus the maximum margin for overfitting is given under the null, hence it is reasonable to expect that it is easier for overfitting to occur (and thus to be detected) under the null. [↑](#footnote-ref-2)
